# Endocrine-Disrupting Organochlorine Pesticides in Human Breast Milk: Changes during Lactation

**DOI:** 10.3390/nu13010229

**Published:** 2021-01-14

**Authors:** Agata Witczak, Anna Pohoryło, Hassan Abdel-Gawad

**Affiliations:** 1 Department of Toxicology, Dairy Technology and Food Storage, Faculty of Food Sciences and Fisheries, West Pomeranian University of Technology in Szczecin, 71-459 Szczecin, Poland; annapohorylo@wp.pl; 2 Applied Organic Chemistry Department, Chemical Industries Research Division, National Research Centre, Dokki, Giza 12622, Egypt; abdelgawadhassan@hotmail.com

**Keywords:** human breast milk, OCPs, infant health, lactation, endocrine disrupting pesticides

## Abstract

The aim of the present study was to assess infant safety associated with the occurrence of endocrine-disrupting organochlorine pesticides (OCP) in breast milk. Moreover, the association between pregnant mothers’ dietary habits and these compounds levels in breast milk was investigated. Breast milk was collected at various stages of lactation. The samples were analyzed by the GC-MS method. The OCP concentrations ranged from < limit of detection (LOD) to 6.81 ng/g lipids. The highest OCP concentrations in breast milk occurred primarily within the first month of lactation, and decreased over the lactation period. It was found that the maternal consumption of certain food products—in particular pork, beef, poultry, eggs, and dairy products—could have affected the content of 1,1’-(2,2,2-Trichloroethane-1,1-diyl)bis(4-chlorobenzene), called DDT and its metabolites in the breast milk. The levels of beta-endosulfan were positively correlated with fish and poultry consumption. The redundancy analysis indicated that the diets of the pregnant women had an important impact on pesticide residues in the breast milk. There is a potential possibility of lowering the content of organochlorine compounds in breast milk by adhering to nutritional recommendations, e.g., avoiding the excessive consumption of fish and other raw food materials of unknown origin.

## 1. Introduction

Breast milk is the first food that humans consume, from birth to a recommended minimum of six months, and it provides all of the necessary nutrients [1]. Breastfeeding is highly beneficial, but breast milk can also transmit endocrine-disrupting organochlorine pesticide (OCP) residues from the mother to the infant [2]. OCPs are characterized by semi-volatility, high environmental persistence, a long half-life, and a high degree of lipophilicity. These properties contribute to adverse effects on human health; among others, this includes endocrine disruption and reproductive toxicity. Long-term exposure to OCP can lead to a variety of endocrine and reproductive disorders, including gonadal changes, changes in reproductive behavior, reduced sperm quality, an increase in organ malformations (e.g., hypospadias), testicular failure (kryptorchim), fetal defects, thyroid dysfunction, and more. The level of thyroid hormones in the prenatal period influences, among others, the proper development and functioning of the peripheral nervous system and the brain. Worryingly, endocrine disrupting compounds (EDCs) can disrupt human endocrine function at very low levels of exposure. It is suspected that EDCs also contribute to the increased incidence of cervical tumors, tumors of the mammary gland and testes, behavioral changes, reduced IQ, and permanent liver and kidney damage. OCPs can act as neurotoxins that block the activity of inhibitory neurotransmitters. They also impair immune functions [3,4]. Organochlorine pesticides (OCPs) have been reported to be associated with an elevated risk of type 2 diabetes in a Chinese population [5].

Infant exposure to OCPs starts in the prenatal period, because of transfusion through the placenta. After birth, OCP exposure continues via lactation. These compounds are accumulated in the mammary glands, and are secreted along with breast milk due to the lipophilicity of OCPs. The increased health risks to infants stem mainly from their incompletely-formed detoxification mechanisms, and the high sensitivity of young organisms. These substances show the ability to penetrate various body barriers, including the blood–placenta barrier, which poses a threat in the prenatal period, increasing the risk of developmental defects. Infants are exposed to high levels of OCPs because of higher food consumption in relation to body weight, that is, several times that of adults [6,7].

Exposure in the postnatal period is also important in terms of the intensive development of organs, e.g., the brain. During the first 90 days of life, the infant develops intensively, and its brain increases in volume by about 64%. Considering the above, EDCs may pose a significant public health risk.

Pregnant women are exposed to these compounds mainly through dietary intake [8], particularly from fish, meat, and milk [9,10].

Despite the decades-long worldwide ban on the use of OCPs, residues are still being found in breast milk all over the world [11,12,13,14]. Since the 1970s, the use of OCPs has been prohibited in Poland due to their high persistence in the environment, toxicity, and ability to accumulate in the food chain of humans and other living organisms. For example, the withdrawal of products with DDT began in Poland in 1976. Organochlorine pesticides—such as DDT, hexachlorocyclohexane (HCH), aldrin, endrin, heptachlor, and hexachlorobenzene (HCB)—have been included in the group of so-called persistent organic pollutants (POPs) due to the Stockholm Convention [15].

Human breast milk is an ideal marker for OCPs, as it provides information on the exposure of mothers and newborns to the toxic effects of these compounds. The accumulation of organochlorine compounds in breast milk can be influenced by many different factors, such as diet, place of residence, smoking, maternal age and weight, and the duration of previous lactation; however, the literature provides divergent information [16].

Therefore, the main purpose of this study was to assess infant safety regarding the content of selected persistent organochlorine pesticides (OCPs) in breast milk during lactation periods. The interest of this study was also the influence of the pregnant mother’s diet on the OCP levels in human breast milk. Correlations were sought between the content of the compounds analyzed and maternal age and body weight, the number of births, the duration of lactation, and the dietary habits of the pregnant women. Based on the surveys conducted, the impact was estimated of the frequency of the consumption of selected food products during pregnancy on the level of pesticides in breast milk.

## 2. Materials and Methods

### 2.1. Participants and the Biological Material Tested

The study participants were 96 mothers, aged 18–36, residing in northwestern Poland. None of the participants had had any previous contact with hazardous chemical compounds at work or in their places of residence. The studies did not include women with contraindications to breastfeeding on the part of the mother or infant, e.g., a severe childhood illness that makes it impossible for the mother to take care of the child, maternal cytotoxic chemotherapy, active maternal tuberculosis, or high-dose alcohol consumption by the mother, etc.

The study was conducted from 2013 to 2015 (a total of 920 samples). The Bioethics Commission at the District Chamber of Physicians in Szczecin consented to the study being conducted (consent of Bioethics Commission No OIL-Sz/MF/KB/452/02/04/2015 of 23 April 2015).

A questionnaire survey was conducted in order to collect the following information about the mothers and their infants: maternal age, number of births, maternal body weight prior to and after pregnancy, infant’s body weight at birth, place of residence, education, and smoking (Table 1). The results from the questionnaire on the basic eating habits of the mothers participating in the study are presented in Table 2. All of the mothers gave the requested information.

Prior to participating in the study, each mother gave written consent for milk collection. Additionally, each participant was informed about the course of the experiment.

The study material analyzed was breast milk. The samples were collected during various lactation periods, ranging from the first to the twelfth month after childbirth. The milk was expressed with breast pumps after the participants had washed their breasts and hands with soap and water [16,17].

All of the mothers were supplied with glass bottles that had been rinsed first with hexane and methanol used for chromatography analysis. The mothers poured the breast milk samples, measuring from 40 to 100 mL, from the breast pumps directly into the glass bottles. The bottles were closed using caps, which were additionally secured inside with aluminum foil. The milk samples were stored at −18 °C in glass bottles in a home freezer until transport to the laboratory.

The milk sampling schedule was the duration of lactation, from the first day after birth to 12 months or more. In the first month of lactation, samples were collected from the mothers weekly, i.e., on days 7, 14, 21, and 28 after delivery. In the following months, the collected milk was the average sample of 4 samples at the end of each month. Each of the mothers participating in the study received precise guidelines on how and when to take the milk. Additionally, each of the mothers was asked to describe the exact time of the milk collection. The more frequent collection of milk in the first month of lactation, compared to the remaining periods, was due to the assumption that there may be the greatest changes in the content of OCPs during this time.

### 2.2. Chemical Materials—Reagents

The reagents (e.g., anhydrous sodium sulfate, hexane, acetone) used for the chromatographic analysis were obtained from Merck.

Standard solutions of the compounds tested were obtained from the following companies:

a) Supelco, USA—4S8913, SS TCL Pesticides;
b) Supelco, USA—N11480 internal standard;
c) Community Bureau of Reference, Belgium—Reference Material BCR 450-PCBs in natural milk powder.

### 2.3. Analytical Methodology

After transport to the laboratory, each sample was labeled with the necessary information regarding the mother and her infant, covered with aluminum foil, and frozen. The samples were homogenized and freeze dried at −60 °C in a LYOLAB 3000 freeze dryer. The lyophilized samples were frozen at −18 °C and stored until the analysis.

The sample preparation included Soxhlet extraction and qualitative and quantitative analyses with gas chromatography-mass spectrometry (GCMS) according to Witczak et al. [18]. The analyses of organochlorine pesticides (aldrin, dieldrin, endrin, alpha-endosulfan, beta-endosulfan, endosulfan sulphate, heptachlor, heptachlor epoxide isomer B and methoxychlor, endrin ketone, endrin, dichlorodiphenyldichloroethylene (pp’-DDE), dichlorodiphenyldichloroethane (pp’-DDD), pp’-DDT, α−HCH, β−HCH, γ−HCH, and δ−HCH) were performed in three replicates using the following gas chromatograph/ mass spectrometer (GC/MS) settings: carrier gas—helium; pressure—0.061 MPa (8.9 psi); flowrate—0.8 mL min^−1^; column (HP-5MS/60.0 m; ID 250 μm, 2.25 μm film thickness of the active phase); oven temperature—start from 90 °C (0.5 min), increase 7 °C min^−1^, 220 °C (12 min), increase 6 °C min^−1^, 285 °C (7 min), increase 5 °C min^−1^, 295 °C (6 min) (post run). The analysis time of one sample was 54.9 min. The detector was a HP 5973 mass spectrometer.

### 2.4. Quality Control

The procedure was evaluated according to Witczak [18]. The monitoring compound recovery, identification, and quantification was based on the following standard solutions: SS TCL Pesticides, Supelco, Bellefonte USA, 4S8913 (aldrin, dieldrin, endrin, alpha-endosulfan, beta-endosulfan, endosulfan sulphate, heptachlor, heptachlor epoxide isomer B, methoxychlor, endrin ketone, endrin, pp’-DDE, pp’-DDD, pp’-DDT, α−HCH, β−HCH, γ−HCH and δ−HCH), and an internal standard (*cis*-chlordane, 80 ng mL^−1^, N11480-10MG, Supelco, USA). The limit of detection (LOD) for each compound was determined according to Commission Directive 2002/63/EC [19]. A blank method was included for every ten samples. The limit of detection (LOD) for each of the compounds tested was 0.01 ng mL^−1^ on average.

The limits of quantitation (*LOQ*) were estimated as (1)

(1) LOQ=10·S

where *S* is the standard deviation of ten independent measurements of the blank sample.

The LOQs varied depending on the pesticide: α−, β−, γ−HCH—0.04 ng mL^−1^; pp’-DDE—0.06 ng mL^−1^; pp’-DDD—0.05 ng mL^−1^; pp’-DDT—0.01 ng mL^−1^; dieldrin, aldrin, heptachlor epoxide—0.06 ng mL^−1^; endrin—0.3 ng mL^−1^; endrin aldehyde, methoxychlor—0.05 ng mL^−1^; heptachlor—0.4 ng mL^−1^; alpha-, beta-endosulfan, endosulfan sulfate, and endrin ketone—0.08 ng mL^−1^. The average recovery of the organochlorine pesticides ranged from 69.9 to 101.3% [18].

### 2.5. Assessment of Infant Exposure Risk

The infant exposure risk was estimated based on the lifetime average daily dose (LADD) and the hazard quotient (HQ) ([20,21,22], taking into account milk consumption, infant body weight, infant sex, and the estimated food intake. Daily milk consumption ranges from 478 mL to 1356 mL, with an average of 798 mL [23,24,25].

In order to assess the health risk, a lifetime average daily dose (LADD) and hazard quotient (HQ) for the pesticides were calculated. The following Equations (2) and (3) were used to estimate the *LADD* and *HQ*:

(2) $$LADD(mg\cdot kg^{-1}\cdot d^{-1})=\frac{C\left(mg\cdot g^{-1}\right)\cdot CR\left(g\cdot d^{-1}\right)}{BW\left(kg\right)}$$

where *LADD* is the daily dosage during life; *C* is the average concentration of the pesticide in human milk, *CR* is the average daily milk consumption, and *BW* is the average body weight in *kg*.

(3) $$HQ=\frac{LADD}{RfD}$$

where *RfD* is the reference dose (*mg·kg^−1^·d^−1^*).

An *HQ* value exceeding 1.0 indicates that it is harmful to human health.

### 2.6. Statistical Analysis

The results obtained were analyzed using Statistica 13.0. ANOVA; the analysis of variance was preceded by Levene’s homogeneity test and the Kolmogorov–Smirnov normality test (K–S test). The results were expressed as arithmetic means with standard deviations (SD) and coefficients of variations (CV). The Pearson correlation coefficient was estimated in order to reflect the relations among the collection time; maternal age; number of births; maternal body weight prior to and after pregnancy; and the milk contents of lipids, protein, lactose, dry matter, and OCPs. Different milk collection times were also taken into account. Guilford’s rule of thumb [26] was used to determine the strength of the relationships. Tukey’s range test (*p* < 0.05) was used to find mean values that were significantly different.

Ordination methods were applied to reveal whether the changes in the OCP contents were driven by the independent variables. Ordination is a noise-reduction technique ([27]. This type of statistical analysis was used because the data set included multiple dependent and independent variables. The set of many variables can be reduced to low-dimensional space.

In this study, redundancy among the predictors was explored with the variance inflation factor (VIF) in canonical correspondence analysis (CCA) [28,29]. Detrended correspondence analysis (DCA) was applied for the dependent variables, and the gradient of the DCA first axis was determined. A redundancy analysis (RDA) on monotonic responses was performed based on this [30,31,32]. A permutation test to assess the significance of consecutive axes was used. The RDA analysis was performed according to The R Project for Statistical Computing [33].

## 3. Results

### 3.1. Characteristics of the Study Participants

The characteristics of the study participants are presented in Table 1. Most of the study participants were women aged 28–32 and 33–36 (Table 1).

### 3.2. Composition of Breast Milk during Lactation

Decreases in the breast milk content of dry matter, lipids, and protein over the lactation period were observed (Table 3), with negative r correlations (*p* < 0.05) of −0.42, −0.38, and −0.50, respectively (Table 3).

### 3.3. OCP Residues in Breast Milk

Residues of organochlorine pesticides were detected in most of the milk samples (Table 4 and Table 5).

**Figure 1:**
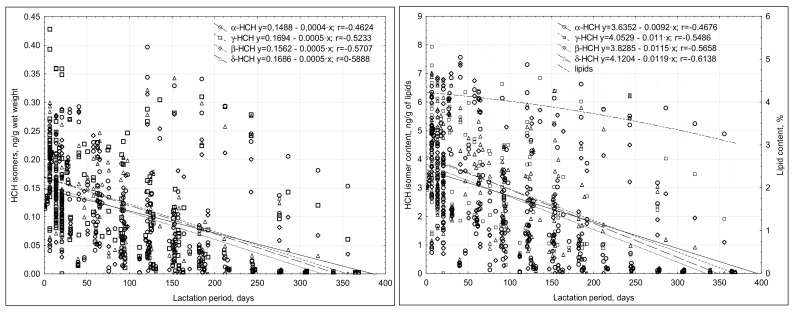
Correlations between the duration of lactation and the concentrations of HCH isomers in the breast milk.

**Figure 2:**
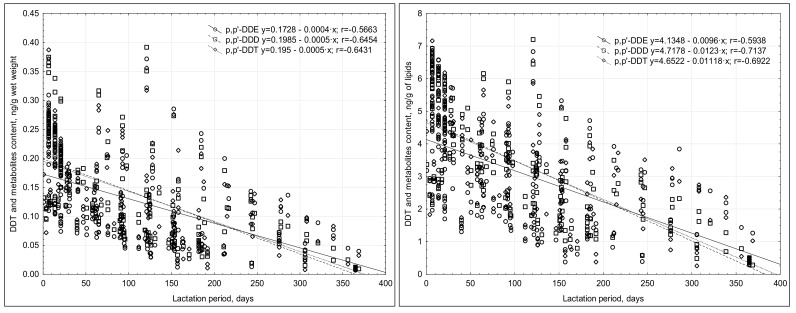
Correlations between the duration of lactation and the concentrations of DDT metabolites in the breast milk.

The concentrations of OCPs noted were low, in the range of <LOQ to 7.5 ng/g lipids. The content of most of the compounds tested decreased throughout the duration of lactation, and a weak negative correlation was noted (Figure 1 and Figure 2). The highest OCP concentrations were usually noted in the breast milk collected in the first month of lactation (Table 4 and Table 5). The analysis of the relationship between the OCP levels and the duration of lactation revealed a negative correlation.

The concentrations of beta-endosulfan and endosulfan sulphate differed significantly in most cases in certain periods of lactation. Weak or moderately-positive correlations of protein content with alpha-endosulfan (r = 0.234) and beta-endosulfan (r = 0.330) were observed.

Among the HCH metabolites, δ-HCH and γ-HCH dominated (Table 5), and the maximum contents of γ-HCH and δ-HCH were noted after seven days of lactation.

**Figure 3:**
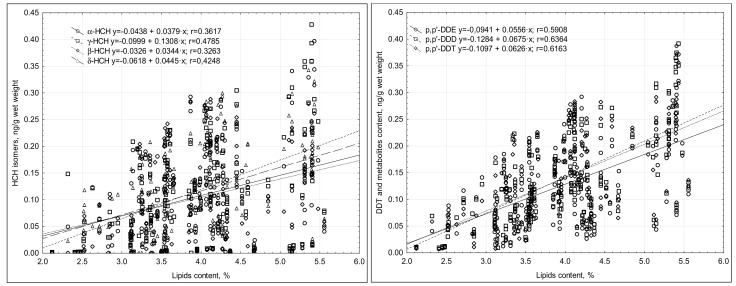
Correlations between the content of HCH isomers and DDT metabolites and the lipid content of the breast milk tested.

The correlation coefficient between the concentrations of HCH isomers and lipid content ranged from 0.30 to 0.48 (Figure 3). The lack of strong correlations indicated that changes in milk fat content were not the only reason for the changes in the concentration of the compounds in the milk’s wet weight.

A significant decrease in DDT metabolite concentrations was noted in the milk during lactation (r = −0.645–0.566) (Table 5), and of the DDT metabolites, p,p′-DDD dominated.

The changes in the content of DDT compounds in the milk’s wet weight depended, to a large extent, on the changes in the lipid content, as was evidenced by the correlation coefficient r being within a range of 0.591 (p,p′-DDE) and 0.636 (p,p′-DDD) (Figure 3).

Additionally, the RDA analysis did not show that maternal age, maternal body weight before pregnancy, or the number of births had a significant effect on the concentration of the compounds analyzed in the breast milk.

### 3.4. Estimating the Infant Health Risk Associated with Breastfeeding

**Figure 4:**
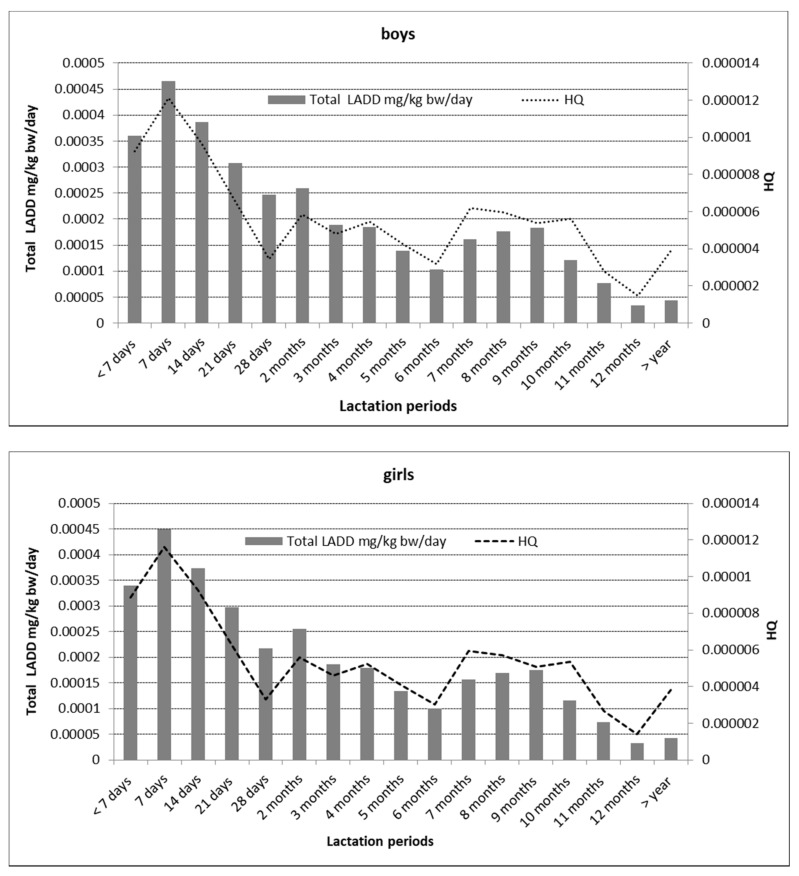
Assessment of infant exposure by sex.

The calculations concerning the volume of ingested milk were based on the literature [23,24,25]. In the assessment of infant health risk, no significant differences were found between the parameters specified for female and male infants. The data from the literature indicate that male infants consume an average of 76 mL more food than female infants. The amount of milk consumed daily by one infant ranges from 478 mL to 1.356 mL, with an average of 798 mL. The LADD ranged from 3.30 × 10^−5^ to 7.84 × 10^−4^ mg/kg bw/day. The highest LADD value was recorded for both female and male infants in the first week of lactation, after which it gradually decreased with the length of the feeding period (Figure 4).

The HQ values were also significantly below 1 and ranged from 1.40 × 10^−6^ to 1.21 × 10^−5^ (Figure 4) [34].

The values of the indicators above signaled that the residues of organochlorine pesticides detected in breast milk posed a low risk to infants, and that the highest uptake of these toxic substances was at the beginning of the breastfeeding period.

### 3.5. Redundancy Analysis (RDA)

The ordination analyses indicated that there were multidimensional relationships among the explanatory and dependent variables. In each case, CCA analysis reduced the number of independent variables, while the DCA analysis resulted in a gradient of the first DCA axis below two standard deviations. Therefore, the RDA method was chosen.

The multidimensional relationships were analyzed between the explanatory variables (lactation period; place of residence; education; maternal age; maternal body weight prior to pregnancy; infant weight; dry milk weight; milk lactose, protein, and lipid content; and maternal habits, such as smoking and fruit, beef, pork, rice, egg, and dairy product consumption) and dependent variables (i.e., concentrations of compounds).

**Figure 5:**
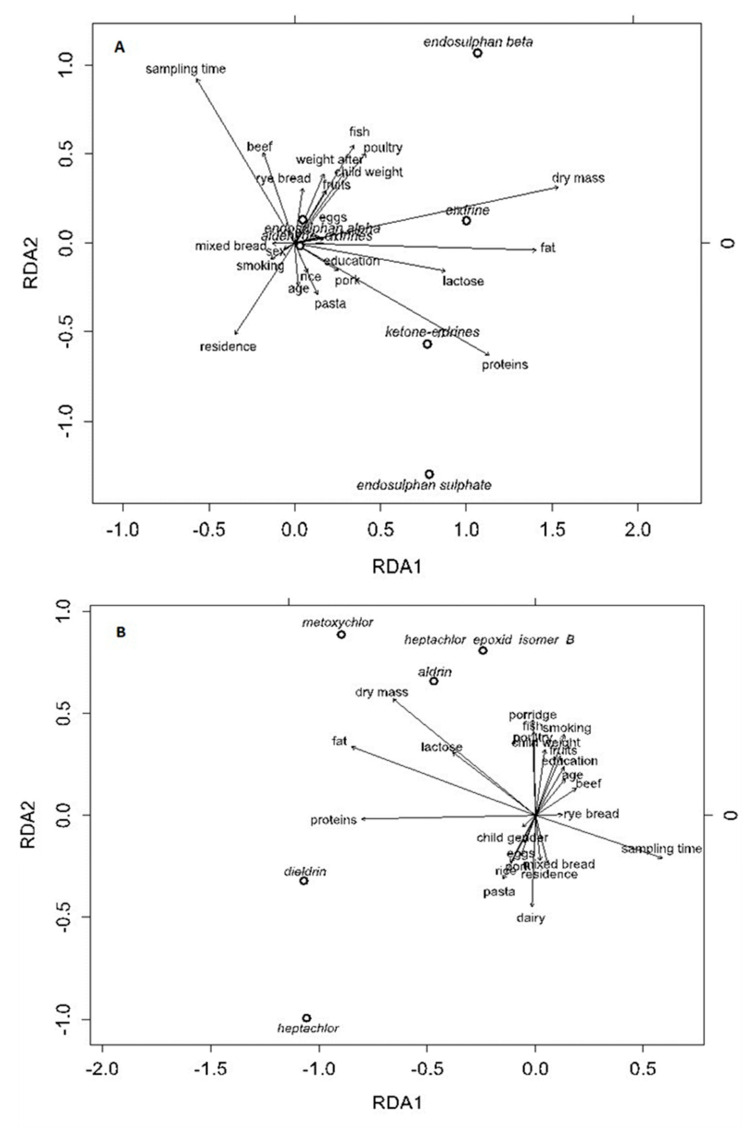

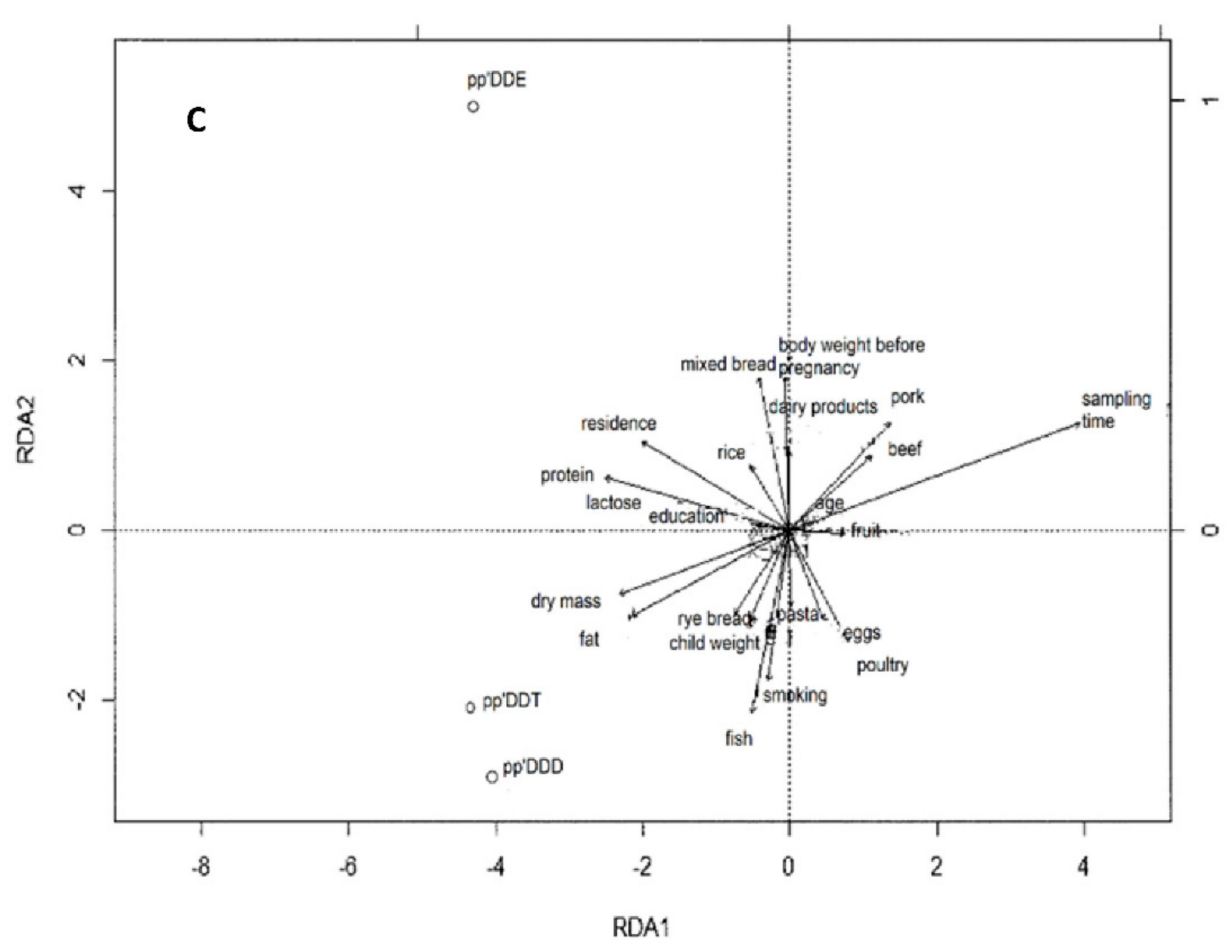
RDA biplot for the relationships between the OCP compounds ((**A**)-endosulfan compounds, endrin compounds, (**B**)–methoxychlor, heptachlor, heptachlor epoxide, aldrin, dieldrin, (**C**)–pp’DDT, pp’DDE, pp’DDD) and the explanatory variables.

The first RDA axis explained 40.9% of the total variance, while RDA2 explained 7.9% of the endosulfan compounds (Figure 5A). Both axes were statistically significant (ANOVA test; *p* < 0.05). The dry mass, fat, protein, and lactose levels made the highest positive contributions to RDA1, while RDA2 was mainly linked to the duration of lactation and the place of residence.

Beta-endosulfan levels were mostly positively correlated with fish and poultry consumption, while the total endosulfan sulphate concentrations decreased over the lactation period. The endrin ketone concentrations increased with protein content, and endrin concentrations correlated positively with dry matter content (Figure 5A).

In Figure 5B, the first RDA axis explained 70.8% of the total variance, while RDA2 explained 14.5%. Both axes were statistically significant (ANOVA test; *p* < 0.05). The results of the RDA analysis (Figure 5B) also indicated that the protein and fat concentrations and milk collection time made the highest positive contribution to RDA1, while RDA2 was mainly linked to the breast milk dry matter content; maternal consumption of dairy, fish and poultry; and maternal smoking. The methoxychlor and aldrin levels were mostly positively correlated with breast milk dry matter and lactose concentrations, but they decreased over the lactation period (Figure 5B).

The RDA analysis did not show any relevant influence of the number of births on the concentrations of the pesticides studied in breast milk. Moreover, no substantial correlation in the levels of toxins determined was found between the milk samples collected from primiparous and multiparous mothers.

Regarding the dependence on milk fat, the first axis RDA1 explained 95.1% of the total variance, while RDA2 explained 2.7% (Figure 5C). Both axes were statistically significant (ANOVA test; *p* < 0.05).

The largest share in the RDA1 axis were factors such as milk intake time, protein, dry matter, and milk fat content. In contrast, the RDA2 axis was predominantly linked to the number of cigarettes that the mothers smoked; the volume of maternal fish, poultry and mixed bread consumption; and maternal body weight before pregnancy (Figure 5C). Both p,p′-DDT and p,p′-DDD were strongly positively correlated with milk fat and dry matter content, but the content of these compounds decreased significantly over the time of milk intake. The RDA analysis also showed negative correlations between p,p′-DDT and p,p′-DDD concentrations and maternal pork and beef consumption. The content of p,p′-DDE in the milk showed a positive correlation with the frequency at which the mothers consumed dairy products and mixed bread, and a negative correlation with their poultry and egg consumption (Figure 5C). However, no significant (*p* < 0.05) correlations were found for HCH isomers. The number of births, maternal weight, and age did not affect the content of HCH isomers in the milk sampled during lactation.

## 4. Discussion

Human milk provides vital protection for and contributes to the development of babies by providing all of the essential nutritional components, such as macronutrients, vitamins, minerals, long-chain polyunsaturated fatty acids, and cytokines. Human milk is recommended as the sole source of nutrition for all babies during the first six months of life. Unfortunately, despite these benefits, human milk also tends to accumulate lipophilic toxins, especially persistent organochlorine pesticides. Their ubiquitous distribution in various environments has a direct effect on human endocrine and reproductive systems. OCPs can reduce fertility and interfere with hormone secretion or activity.

In the light of the health risk to infants, it is important to test for the presence of organochlorine pesticides in breast milk, and to look for the correlation between the presence of these compounds and the age of the mothers, body weight, number of births, duration of lactation, and nutritional habits associated with natural feeding.

The residues of the pesticides (OCPs) analyzed in human milk were at different levels. The literature data show a wide range of pesticide residues in breast milk, depending on the country.

Overall, the ED pesticides in human milk have decreased over the past 30 years in many countries. For example, for DDT, there has been a decrease from 2000–32,000 ng/g lipids to 0.1 ng/g lipids since the 1990s. [35,36]. Based on the results obtained in this study, it can be concluded that there is a similar tendency in Poland [37].

For example, other studies reported the content of aldrin ranging between 0.009 and 40 ng/g lipids, and dieldrin ranging between 1 and 713 ng/g lipids [11,12,13,16,38]. In light of these data, the values obtained in the current study were low. Higher dieldrin concentrations in breast milk were also reported by many authors in different countries, such as Australia and New Zealand [12,39]. The high dieldrin levels in breast milk collected from mothers in different countries could have resulted from its persistence in the natural environment and its ability to bioaccumulate.

In contrast to the present study, Croes et al. [16] did not report either heptachlor or heptachlor epoxide isomer B in breast milk collected from rural Belgian mothers. Mueller et al. [39] found heptachlor-epoxide isomer B in breast milk collected between the second and eighth weeks of lactation in samples from rural and urban Australia, and the levels of this averaged 16.7 ng/g lipids in the samples from rural areas, while the average in samples from urban areas was 2.21 ng/g lipids. These values were significantly higher compared to the results obtained in our study. Lower concentrations of heptachlor (0.013 ng/g lipid) and heptachlor epoxide isomer B (0.456 ng/g lipid) were reported in the breast milk samples collected from women residing in New Zealand [12].

Studies conducted from 2007 to 2008 in Turkey by Çok et al. [13] confirmed the presence of alpha-endosulfan and beta-endosulfan in breast milk at levels that were considerably higher compared to those found in the present study. These authors observed similar levels of alpha-endosulfan in both primiparous and multiparous mothers, and they reported beta-endosulfan residue averages of 38 ng/g lipids. Similar findings have been reported by many authors [12,14].

Not until 2010 were α-HCH, β-HCH, and lindane included among the substances regulated by the Stockholm Convention, which resulted in a total ban on their production and use. The contents of this pesticide in human milk were recorded in 2000–2004 in Norway (14 ng/g lipids) and Sweden (10 ng/g lipids) [40,41].

The presence of HCH in human milk can result from the incorrect use of pesticide preparations, and from noncompliance with waiting periods in the years before their use was banned. A secondary effect of the occurrence of these compounds in the environment is their accumulation in the raw materials used in foods that are consumed by future mothers. Bulut et al. [42], Fromberg et al. [43], Witczak [18], and Witczak and Pohoryło [44] all reached this conclusion in their studies on the presence of total HCH in cow’s milk, sheep’s milk, butter, and cheese. Of all isomers, β-HCH was noted most commonly, which is a result of its high stability and persistence. In the human body, the β-isomer is usually present in higher concentrations than those of α- and γ-HCH, which metabolize into β-HCH [45]. The β-HCH content in the present study (average 2.03 ng/g lipids) corresponds to the results of Croes et al. [16] and Klinčić et al. [46] in Belgium and Croatia, respectively. Both teams of researchers found the content of this isomer to be in the range of 0.6–9.0 ng/g lipids. The dominance of β-HCH in human milk can be affected by a number of mechanisms that modify the chemical structure of these compounds. For example, in the environment, some bacteria can slowly isomerize lindane into α-, β- and δ-HCH, mainly under anaerobic conditions. HCH degradation is slow, and the efficiency of these processes under anaerobic conditions is greater than in aerobic conditions. The high content of these compounds can result from their presence in the environment, as a result of the long-term use of HCH to fight malaria or typhoid, and because they are highly stable and resistant to degradation [8,47]. The highest HCH value reported was in the milk of women in India in 2000–2012 (35,000 ng/g lipids) [48]. Such a high level of this pesticide could be because India was one of the leading producers and consumers of chlorinated pesticides, particularly of HCH, until the ban/restriction of their use in the late 1990s. However, a significant proportion of these compounds are still permitted for use in malaria control.

A large discrepancy can be noted when our results are compared with the data available in the literature. Polder et al. [49] demonstrated that milk consumed by infants is completely safe; however, Müller et al. [38] reached different conclusions. These authors reported that the tolerated daily intake of total DDT was exceeded in human milk samples from Northern Tanzania. Gebremichael et al. [50] reported that Ethiopia had one of the largest stocks of obsolete pesticides in Africa. Exposure to high concentrations of DDT compounds can have many negative health consequences. In Poland, the content in breast milk of DDT metabolites has decreased over the years [37]. In the light of the preceding data, the average total DDT content determined in the current study was low (8.14 ng/g lipids). The significant reductions in the total DDT content could have resulted from the restrictive provisions of the total ban on the use of plant protection products not only in the Stockholm Convention [15] but also from those imposed in all European Union countries. As in the case of HCH isomers, studies have shown that expectant mothers’ consumption of cow’s milk and milk products might be one of the ways in which they are exposed to DDT [18]. The main source of organochlorine compounds for dairy animals is contaminated feed [51]; the degree that DDT metabolites transfer into milk ranges from 4% (p,p′-DDT) to 80% (p,p′-DDE) [52].

In this study, the concentration of selected organochlorine pesticides in the mother′s milk may have been associated with the individual features of the mother and her infant, as well as their dietary habits.

In our research, the RDA analysis did not show that maternal age, maternal body weight before pregnancy, or the number of births had a significant effect on the concentration of the compounds analyzed in the breast milk.

In relation to maternal age, similar results were obtained by Hassine et al. [8] for HCH isomers, and by Behrooz et al. [53] and Azeredo et al. [54] for DDT and its metabolites. The lack of correlation between the content of organochlorine pesticides and the age of the mothers may result from too-short an exposure period, or the disappearance of these compounds in the environment. However, some authors have noted various correlations between the concentration of these pesticides in milk and the age of the mothers, positive [55,56] or negative [46]. The increase in the concentration of organochlorine pesticides with the age of mothers may also be the result of women′s eating habits. It has been proven that pesticides are resistant to metabolic processes in the body, and that they bioaccumulate with age [57,58]. The studies by Dimitriadou et al. [56] and Müller et al. [38] brought similar conclusions to ours: the body weight of the women, both before and after pregnancy, influenced the changes in the concentration of these compounds in the milk. The redundancy analysis carried out in this study confirmed that the content of organochlorine pesticides in human milk was not influenced by the sex of the child, which was also confirmed in [38,56]. An interesting relationship was also observed by Croes et al. [16]. The authors proved that the level of ∑DDT in human milk increased with the increase in the consumption of dairy products by future mothers. Our research, however, did not confirm a similar relationship.

This assessment of infant exposure to the residues of selected OCPs in human milk is the first study of its kind in northwestern Poland.

The risk associated with the exposure to OCPs depends on the dose, the duration of exposure, and whether or not the acceptable daily intake (ADI) is exceeded. This is important because infants consume many times more food per kilogram of body weight than do adults; they are in a phase of dynamic growth and development, and their exposure to the toxic effects of EDCs is much higher.

The conducted risk analysis allowed for the evaluation of the tested food as safe for infants. It has also been shown that, with the increasing period of lactation, the content of organochlorine pesticides in milk decreases, with the largest pool of DDT and HCH being accumulated in the milk by the infant in the first weeks of life. This is a valuable observation which is also important from a clinical point of view. Attention was also paid to the potential possibility of lowering the content of organochlorine compounds in breast milk by following specific nutritional recommendations for women expecting a child, e.g., avoiding the excessive consumption of fish, especially of unknown origin, and eliminating smoking. The diet of pregnant women may significantly affect the concentration of OCPs in human milk, because they take up these compounds mainly from food. The highest residues of these compounds was found in fish, meat, poultry, eggs, milk, and dairy products, as well as in vegetable oils, nuts, avocado, sesame, or olives [59,60,61]. This is due to the lipophilicity, persistence, and tendency to cumulation of these compounds, both in plant and animal organisms.

## 5. Conclusions

In the breast milk, a decrease in the content of the analysed OCPs, as well as the dry matter, lipids, and protein over the lactation periods analysed were observed. Moreover, our research showed that the highest OCP concentrations in breast milk occurred primarily within the first month of lactation.

The RDA analysis indicated that the type of diet that pregnant women consume has an important impact on the pesticide residue levels in their breast milk. Among others, the concentration of beta-endosulfan residues in breast milk correlated positively with the frequency of fish and poultry consumption by pregnant women. It was found that the maternal consumption of certain food products, in particular pork, beef, poultry, eggs, and dairy products, could have affected the content of DDT and its metabolites in the breast milk. The levels of beta-endosulfan were primarily positively correlated with maternal fish and poultry consumption. Maternal age, place of residence, and infant birth weight were not correlated with the DDT or HCH concentrations in breast milk.

Current Polish law does not specify the maximum acceptable limits of OCPs in breast milk; therefore, any assessment of breast milk safety regarding OCPs must be made in reference to the regulations on food for infants and young children. The levels of OCP residues in the breast milk examined in this study were below the maximum residue levels (MRL) (set forth in Polish Journal of Laws no. 180 item 1214 of 16 September 2010 [62] and Polish Journal of Laws item 1026 of 16 June 2015 [63].

The analysis of the organochlorine pesticide residues in breast milk indicated that the values of the parameters used to assess the infant risk were very low. The assessment of infant health risk indicated that there were no significant differences for female or male infants between parameters such as LADD and HQ. A gradual decrease was observed in LADD over longer breastfeeding periods. The HQ values were also low, at less than 1. The analysis of the results of this study led to the conclusion that mothers’ milk, although it is not devoid of pesticide residues, is safe for infants. Despite the presence of organochlorine impurities in breast milk, the risk assessment parameters proved that breast milk is safe for infant health. However, we would like to emphasize the need for the continuous monitoring of endocrine-disrupting organochlorine xenobiotics in human milk. This is particularly important because of the lack of legal regulations on this matter. There is also a potential possibility of lowering the content of organochlorine compounds in breast milk by adhering to certain nutritional recommendations for pregnant women, e.g., avoiding the excessive consumption of fish and other raw food materials of unknown origin.

## Figures and Tables

**Table 1:** Characteristics of the pregnant women participating in the study (n^a^ = 96, 920 samples of milk)**.**

No.	Basic Information on Mother and Child	Response Given in Questionaire	Percentage of Given Responses (%)
1.	mother’s age [years]	<18	4.2 (%)
		18–22	-
		23–27	25 (%)
		28–32	37.5 (%)
		33–36	33.3 (%)
		>36	-
2.	body weight prior pregnancy [kg]	<55	25 (%)
		56–60	12.5 (%)
		61–65	20.8 (%)
		>65	41.7 (%)
3.	body weight in the first week after the childbirth [kg]	55–60	20.8 (%)
		61–65	20.8 (%)
		66–70	25 (%)
		>70	33.4 (%)
4.	place of residence	city > 5.000 inhabitants	83.3 (%)
		town < 5.000 inhabitants	4.2 (%)
		Country	12.5 (%)
5.	education	Primary	4.2 (%)
		Secondary	-
		Higher	95.8 (%)
6.	number of given childbirths	1	62.5 (%)
		2–3	37.5 (%)
		4–5	-
		>5	-
7.	type of birth	natural forces	50 (%)
		C-section	50 (%)
8.	infant’s sex	Boy	62.5 (%)
		girl	37.5 (%)
9.	infant’s body weight in the first week [kg]	2.4–2.8	4.2 (%)
		2.9–3.2	45.8 (%)
		3.3–3.6	45.8 (%)
		3.7–4.0	-
		>4.0	4.2 (%)
10.	smoking	never smoked	75 (%)
		quitted before pregnancy	16.7 (%)
		used to smoke (number of packs á 20 cigarettes) ➢ up to one a day ➢ more than one a day	4.2 (%) 4.2 (%)
		smokes at present (number of packs) ➢ up to one a day ➢ more than one a day	- -

n^a^—number of mothers participating in the study.

**Table 2:** Eating habits of the pregnant women based on the survey.

Product		Never	Less that Once a Week	Once a Week	Twice a Week	More Than Twice a Week	Every Day
		Percentage of Responses [%]					
Fish		4.2	37.5	29.2	12.5	16.6	-
Dairy products		12.5	-	-	-	25	62.5
Meat	beef	16.7	58.3	8.3	12.5	4.2	-
	pork	-	8.4	20.8	33.3	37.5	-
Poultry		-	-	8.3	16.7	70.8	4.2
Eggs		-	29.1	12.5	12.5	41.7	4.2
Fruit		-	-	4.2	4.2	16.6	75
Vegetables		-	-	-	4.2	8.3	87.5
Cereal products	groats	-	50	25	20.8	4.2	-
	rice	-	37.5	50	12.5	-	-
	pasta	-	29.2	41.6	29.2	-	-
	whole meal bread	8.3	16.7	16.7	8.3	20.8	29.2
	wheat bread	8.3	12.5	12.5	8.3	25	33.4
	mixed bread	12.5	25	20.8	8.3	29.2	4.2

**Table 3:** Mean content of the basic composition of breast milk (number of samples *n* = 920).

Lactation Period	Number of Samples	Protein [%]	Lactose [%]	Dry Matter [%]	Lipid [%]
<7 days	17 ^a^	1.49 ± 0.23 ^b^	5.33 ± 0.30	15.34 ± 0.00	3.92 ± 0.00
7 days	26	1.48 ± 0.18	7.27 ± 0.47	13.21 ± 1.38	4.60 ± 0.77
14 days	59	1.28 ± 0.26	6.90 ± 0.24	12.71 ± 0.79	4.34 ± 0.59
21 days	98	1.18 ± 0.24	6.77 ± 0.29	12.12 ± 1.05	4.13 ± 0.63
28 days	39	1.07 ± 0.30	6.98 ± 0.68	12.54 ± 0.56	3.48 ± 0.36
2nd month	75	1.12 ± 0.24	6.87 ± 0.34	12.50 ± 1.41	4.22 ± 0.86
3rd month	75	1.07 ± 0.21	6.81 ± 0.32	12.04 ± 1.32	3.85 ± 0.76
4th month	92	1.08 ± 0.22	6.83 ± 0.34	12.08 ± 1.86	3.90 ± 0.77
5th month	88	1.11 ± 0.28	6.84 ± 0.25	12.04 ± 1.74	3.73 ± 0.88
6th month	88	1.07 ± 0.25	6.66 ± 0.27	11.36 ± 1.36	3.67 ± 0.90
7th month	69	1.19 ± 0.25	6.84 ± 0.33	12.36 ± 1.95	4.18 ± 0.89
8th month	39	1.17 ± 0.17	6.92 ± 0.26	13.52 ± 1.70	4.10 ± 0.60
9th month	29	0.99 ± 0.06	6.85 ± 0.30	12.36 ± 0.88	4.22 ± 0.23
10th month	29	1.08 ± 0.15	6.91 ± 0.05	10.57 ± 0.58	3.83 ± 0.47
11th month	29	0.93 ± 0.04	6.62 ± 0.43	10.03 ± 1.14	3.49 ± 0.03
12th month	29	0.84 ± 0.06	6.54 ± 0.25	10.44 ± 0.57	3.14 ± 0.02
>year of breastfeeding	39	0.88 ± 0.03	6.45 ± 0.08	8.66 ± 0.25	2.37 ± 0.17
mean content		1.12 ± 0.18	6.73 ± 0.30	11.99 ± 1.09	3.83 ± 0.52

^a^ Each sample was analyzed in triplicate analytical duplicates; ^b^ arithmetic average ± standard deviation.

**Table 4:** Mean content of selected organochlorine pesticides in breast milk.

Lactation Period	Aldrin	Dieldrin	Endrin	Endrin Aldehyde	Endrin Ketone	Heptachlor	Heptachlor-Epoxide, Isomer B	Methoxychlor	Alpha-Endosulfan	Beta-Endosulfan	Endosulfan Sulfate
	ng/g lipids										
<7 days	4.13 ± 2.36 ^a^	5.19 ± 2.68	5.69 ± 4.52	<LOQ ^a^	1.27 ± 0.67	2.04 ± 1.07	2.53 ± 1.05	7.52 ± 2.89	0.399 ± 0.281	5.60 ± 5.46	2.62 ± 1.36
7 days	3.59 ± 1.61	6.36 ± 2.74	5.48 ± 2.20	0.497 ± 1.127	4.13 ± 2.23	6.63 ± 3.46	2.33 ± 2.47	6.81 ± 1.10	0.623 ± 1.010	2.73 ± 2.80	5.65 ± 2.75
14 days	2.60 ± 1.16	5.59 ± 2.21	3.85 ± 1.92	0.123 ± 0.408	3.56 ± 2.10	6.60 ± 2.44	1.55 ± 1.97	6.29 ± 1.43	0.354 ± 1.161	1.83 ± 2.22	4.68 ± 3.01
21 days	1.97 ± 1.28	3.89 ± 2.11	2.61 ± 1.76	<LOQ	2.43 ± 1.98	5.77 ± 2.28	1.13 ± 2.34	5.12 ± 1.77	0.061 ± 0.182	1.22 ± 1.68	3.48 ± 2.17
28 days	1.59 ± 0.51	2.08 ± 1.29	2.23 ± 1.17	<LOQ	1.28 ± 1.25	3.04 ± 2.27	0.477 ± 0.900	5.63 ± 1.76	0.057 ± 0.114	1.38 ± 1.59	2.83 ± 2.42
2nd month	1.73 ± 1.50	3.16 ± 2.39	3.40 ± 2.91	0.545 ± 1.317	1.78 ± 2.11	4.01 ± 2.06	0.990 ± 1.270	5.20 ± 2.46	0.524 ± 1.945	3.14 ± 5.07	3.18 ± 1.87
3rd month	1.43 ± 1.10	3.00 ± 2.63	3.10 ± 2.78	0.200 ± 0.554	1.07 ± 1.27	3.11 ± 1.28	1.26 ± 2.18	4.52 ± 2.83	0.147 ± 0.532	1.91 ± 2.13	3.23 ± 2.13
4th month	1.59 ± 1.38	3.01 ± 3.10	3.03 ± 3.19	0.198 ± 0.791	2.00 ± 2.91	3.15 ± 2.17	1.10 ± 1.70	5.00 ± 2.84	0.052 ± 0.209	1.68 ± 2.10	2.59 ± 2.04
5th month	1.13 ± 0.95	2.79 ± 2.83	2.60 ± 2.78	<LOQ	1.11 ± 1.57	2.20 ± 4.14	0.701 ± 1.314	4.14 ± 2.26	0.065 ± 0.235	1.59 ± 1.83	2.19 ± 1.63
6th month	0.61 ± 1.36	2.40 ± 2.24	1.59 ± 2.14	<LOQ	1.14 ± 2.57	1.69 ± 1.53	0.091 ± 0.328	3.64 ± 2.18	0.083 ± 0.299	1.79 ± 5.21	1.59 ± 2.10
7th month	1.23 ± 1.64	3.73 ± 2.45	4.06 ± 3.61	<LOQ	1.98 ± 2.59	3.66 ± 2.51	0.028 ± 0.075	5.18 ± 1.73	0.305 ± 0.806	3.08 ± 4.12	2.75 ± 2.42
8th month	0.63 ± 0.60	4.07 ± 3.01	4.21 ± 2.49	<LOQ	3.87 ± 2.54	3.72 ± 1.82	0.071 ± 0.142	5.88 ± 1.73	<LOQ	4.85 ± 4.22	2.23 ± 1.80
9th month	1.42 ± 0.71	1.70 ± 2.27	5.25 ± 4.63	<LOQ	1.39 ± 2.41	3.02 ± 0.56	0.063 ± 0.039	4.90 ± 0.51	<LOQ	3.34 ± 2.21	1.51 ± 1.24
10th month	0.82 ± 0.71	5.23 ± 3.93	2.94 ± 1.25	<LOQ	1.15 ± 1.99	4.14 ± 4.83	<LOQ	5.13 ± 4.03	<LOQ	2.01 ± 1.90	3.89 ± 4.89
11th month	0.49 ± 0.37	2.51 ± 0.90	1.53 ± 0.69	<LOQ	0.282 ± 0.489	4.09 ± 4.74	0.154 ± 0.267	3.32 ± 2.23	<LOQ	1.205 ± 1.072	2.18 ± 3.06
12th month	0.074 ± 0.064	1.77 ± 0.71	0.14 ± 0.32	<LOQ	<LOQ	3.09 ± 3.87	<LOQ	1.10 ± 1.57	<LOQ	<LOQ	0.297 ± 0.288
> year of lactation period	1.21 ± 2.10	5.63 ± 2.61	2.03 ± 2.19	<LOQ	0.954 ± 1.909	2.70 ± 2.73	<LOQ	3.23 ± 1.71	<LOQ	<LOQ	<LOQ

LOQ—limit of quantitation of organochlorine pesticides (OCPs); ^a^ arithmetic average ± standard deviation.

**Table 5:** Mean content of selected organochlorine pesticides in breast milk—continued.

Lactation Period	α-HCH	γ-HCH	β-HCH	δ-HCH	*p,p′*-DDE	*p,p′*-DDD	*p,p′*-DDT
	ng/g lipids						
<7 days	3.15 ± 0.12 ^a^	3.13 ± 0.30	3.19 ± 0.26	3.18 ± 0.26	0.115 ± 0.013	0.118 ± 0.020	0.132 ± 0.057
7 days	4.05 ± 1.45	4.53 ± 1.72	4.36 ± 1.53	4.68 ± 1.80	0.221 ± 0.065	0.245 ± 0.080	0.239 ± 0.072
14 days	3.71 ± 0.962	4.01 ± 1.65	3.80 ± 1.53	4.37 ± 1.49	0.186 ± 0.056	0.208 ± 0.066	0.203 ± 0.057
21 days	3.25 ± 1.06	3.43 ± 2.17	3.24 ± 1.95	3.57 ± 1.50	0.161 ± 0.044	0.189 ± 0.052	0.185 ± 0.042
28 days	3.71 ± 1.93	4.01 ± 1.79	4.08 ± 2.23	3.92 ± 2.06	0.140 ± 0.027	0.147 ± 0.022	0.159 ± 0.011
2nd month	2.88 ± 2.06	3.30 ± 1.80	2.88 ± 2.06	3.63 ± 1.57	0.138 ± 0.054	0.162 ± 0.059	0.159 ± 0.057
3rd month	2.54 ± 1.10	2.95 ± 1.51	2.41 ± 1.88	3.14 ± 1.44	0.111 ± 0.051	0.126 ± 0.055	0.127 ± 0.054
4th month	2.54 ± 2.19	2.70 ± 2.08	2.15 ± 1.99	2.50 ± 1.53	0.111 ± 0.075	0.125 ± 0.079	0.121 ± 0.076
5th month	2.00 ± 1.86	2.22 ± 1.53	1.94 ± 1.91	2.13 ± 1.74	0.085 ± 0.069	0.094 ± 0.064	0.101 ± 0.071
6th month	1.56 ± 1.16	1.75 ± 1.92	1.42 ± 1.07	1.37 ± 1.31	0.066 ± 0.060	0.072 ± 0.049	0.074 ± 0.051
7th month	1.67 ± 1.19	1.39 ± 1.99	1.37 ± 1.09	1.24 ± 1.82	0.088 ± 0.072	0.088 ± 0.050	0.082 ± 0.055
8th month	1.63 ± 2.49	1.70 ± 1.78	1.09 ± 1.84	1.76 ± 1.74	0.097 ± 0.057	0.086 ± 0.048	0.082 ± 0.049
9th month	1.00 ± 1.56	0.814 ± 1.28	0.61 ± 0.87	1.04 ± 1.66	0.082 ± 0.026	0.068 ± 0.016	0.071 ± 0.044
10th month	1.98 ± 3.31	1.39 ± 2.28	0.98 ± 1.56	0.44 ± 0.64	0.094 ± 0.042	0.062 ± 0.021	0.076 ± 0.037
11th month	1.78 ± 2.96	1.20 ± 1.97	0.65 ± 1.11	0.34 ± 0.50	0.067 ± 0.023	0.039 ± 0.013	0.050 ± 0.025
12th month	1.65 ± 1.80	0.661 ± 1.08	0.37 ± 0.33	0.017 ± 0.023	0.036 ± 0.011	0.016 ± 0.008	0.019 ± 0.012
> year of lactation period	0.054 ± 0.009	0.070 ± 0.015	0.031 ± 0.008	0.049 ± 0.013	0.009 ± 0.001	0.010 ± 0.001	0.009 ± 0.002

^a^ Arithmetic average ± standard deviation.

## Data Availability

The data presented in this study are available on request from the corresponding author.
